# Flax Fibre Yarn Coated with Lignin from Renewable Sources for Composites

**DOI:** 10.3390/polym14194060

**Published:** 2022-09-27

**Authors:** Claudia Möhl, Timo Weimer, Metin Caliskan, Tom Hager, Stephan Baz, Hans-Jürgen Bauder, Thomas Stegmaier, Werner Wunderlich, Götz T. Gresser

**Affiliations:** 1 German Institutes of Textile and Fibre Research (DITF), Körschtalstrasse 26, 73770 Denkendorf, Germany or; 2 Institute for Textile and Fibre Technologies (ITFT), University of Stuttgart, Pfaffenwaldring 9, 70569 Stuttgart, Germany

**Keywords:** flax yarn, lignin, bio-based thermoplastics, bio-based matrix material, yarn coating, extrusion, natural fibre, composite

## Abstract

The present experimental work analyses the potential of lignin as a matrix for materials made from renewable resources for composite components and the production of hybrid semi-finished products by coating a flax fibre yarn. Natural fibres, due to their low density, in combination with lignin can be a new renewable source for lightweight products. For this purpose, the extrusion process was adapted to lignin as a matrix material for bio-based composites and coating of natural fibre yarns. A commercial flax yarn is the basis for the lignin coating by extrusion. Subsequently, the coated flax yarn was characterised with regard to selected yarn properties. In order to produce composite plates, the lignin-coated flax yarn was used as warp yarn in a bidirectional fabric due to its insufficient flexibility transversely to the yarn axis. The commercial flax yarn was used as weft yarn to increase the fibre volume content. The tensile and flexural properties of the bio-based composite material were determined. There was a significant difference in the mechanical properties between the warp and weft directions. The results show that lignin can be used as matrix material for bio-based natural fibre composites and the coating of natural fibre yarns is an alternative to spun hybrid yarns.

## 1. Introduction

Using plant and animal resources for clothing, everyday objects, and tools is as old as humanity itself. Wood, resins, fibres, leather and other naturally available materials were used to construct items that contributed significantly to the survival and development of the human species. This changed only when petroleum-based polymers and modern fibre-reinforced composites were developed. Favoured by low production costs due to the parallel introduction of mass production and adapted properties, demand for polymers and modern fibre-reinforced composites grew exponentially. Between 2010 and 2019, global plastics production increased by around 100 million tonnes to 368 million tonnes to meet the needs of construction, automotive, aerospace, textile, packaging, pharmaceutical and many other industries [1,2]. The consequences of the extensive use and the “take, make and dispose” approach include overflowing landfills, improper disposal in forests, rivers and oceans, contamination of soils and water bodies, the release of greenhouse gases, air pollution from incineration, and threats to biodiversity and mankind itself [3,4]. In this context, the research and development of fully bio-based fibre-reinforced composites that have a lower environmental impact from production to disposal has become an important and pressing goal of our time. Therefore, in this study, the suitability of lignin as a thermoplastic matrix from renewable resources is evaluated and an alternative semi-finished product manufactured by coating flax fibre yarns is investigated.

Lignin is the second most abundant polymer in nature after cellulose. Lignin is an amorphous, three-dimensional, cross-linked heteropolymer, it always consists of the same three main phenylpropane units (guaiacyl, syringyl and p-hydroxyphenyl), which, however, occur in different proportions depending on the biological influencing factors and the starting material used [5]. The annual production volume of lignin from the pulp and paper industry is around 70 million tonnes worldwide, which would make its use possible for various industrial applications. However, it is difficult to develop higher-quality materials from lignin [6]. The heterogenic and the aromatic structure with an even distribution of the molecular weight of the lignin leads to a poor solubility in most known organic solutions, because lignin sterically prevent the reactive sites [7]. That explains why only 2% of lignin produced is currently used as a source for chemicals or materials, while the rest is burned for energy production [8]. A new approach to solving lignin and making lignin processable and usable for products is the use of ionic liquids. Lignin can be dissolved in ionic liquids and the ionic liquids is also reusable [5]. Lignin, with its special properties such as high thermal stability, high carbon content, good ultra violet absorption, low material costs, and above all, biodegradability, is a potential sustainable component for bio-based composites [9,10]. Therefore, lignin was researched years ago as an additive for polymers [11,12,13], as a reinforcing component in composite materials [14] and as a thermoplastic as well as a thermosetting matrix material [15,16,17]. It has been used in various applications such as biosurfactant, antimicrobial agents, active packaging, activated carbon, and supercapacitor make this abundant material promising low-cost additive [18]. Recently, research on lignin has focused more on its use as a raw material for bio-based carbon fibres and on the production of biodegradable composites from renewable raw materials.

In addition to the properties already mentioned, blending lignin with other biopolymers is attractive, because of the variety of modifications possible due to its chemical structure [19]. Therefore, many studies on the production of bioplastics focus on the in-corporation of lignin into natural biopolymers such as polylactic acid (PLA) [20], polyhydroxybutyrate (PHB) [21] and polybutylene succinate (PBS) [22]. The addition of lignin as a reinforcing material usually lowers costs and water absorption [21]. In addition, lignin has significant antioxidant properties, as the phenolic hydroxyl groups can scavenge free radicals [23]. The compatibility between cellulose and other hydrophobic biopolymers could be greatly enhanced by lignin with its polar phenolic hydroxy groups and non-polar hydrocarbon groups [24]. The addition of lignin could improve the compatibility between cellulose and matrix [25,26]. In [24], it is described that impact strength decreased after the addition of lignin, while Young’s modulus and tensile strength improved significantly. In cellulose-lignin bio-composites, the cellulose-containing component enhances the mechanical strength of the composites, while lignin improves the thermal stability of the polymer matrix and ensures good dispersion of the cellulose in the biopolymers [27,28].

By using fully bio-based composites, the ecological disadvantages of glass fibre reinforced plastics and carbon fibre reinforced plastics can be eliminated and, as an alternative, equally high-performance and durable fibre composite components can be produced. Compared to carbon and glass fibres, natural fibres are renewable, sustainable, do not splinter and consume less energy during production [29]. In addition, natural fibres offer high potential for lightweight construction and good insulating properties due to their low density [30].

Yarn coating is used in the textile industry to enhance the functional properties of yarns. One field of application is the sizing of yarns. In this process, the yarn is covered with a starch or synthetic component-based coating to protect the yarn from yarn breakage and production losses in subsequent processing operations such as weaving. The yarn coating is used when an additional functional feature is imparted to the yarn as an end product or when finishing the end product is not possible. Special functions such as abrasion resistance, flame retardancy, antibacterial properties, dirt repellence, electrical conductivity, UV protection can be added. Coated yarns serve for special niche markets, such as sewing yarns, used in fly screens, cord and more technical applications.

Often, the mechanical yarn characteristics and the sensitivity against moisture prevents the use of natural fibre yarn in composites [31]. Researchers are already working on fibres and yarns coated with matrix material for the production of thermoplastic fibre composite materials [32]. The disadvantages of the natural fibre yarns can be improved or adapted to the respective area of application by means of a targeted yarn coating for the adaptation of the yarn properties and the extension of the yarn functionality. This can be very different in terms of process technology, adapted to the respective application of the yarn. While classic aqueous coatings are mostly applied by dipping and squeezing systems, thermoplastic granulates are extruded and applied to the yarn via nozzles. The desired yarn properties have a wide range and are determined by the subsequent application. The functional coatings can be applied individually according to customer requirements.

A thorough analysis of the literature shows that lignin is mainly used as a filler or in small quantities as an admixture for thermoplastic polymers. It is currently used as a main component in thermoplastics exclusively in the form of granules for injection moulding and extrusion applications. The present experimental study investigates lignin as a coating for natural fibre yarns and its utilisation in textile processes to produce a semi-finished textile product for sustainable fibre reinforced plastic applications.

## 2. Materials and Methods

### 2.1. Materials

For this feasibility study, commercially available flax yarn from N.V. JOS VANNESTE S.A., Harelbeke, Belgium, and ARBOFORM^®^ L V100 from TECNARO GmbH, Ilsfeld, Germany were acquired. ARBOFORM^®^ L V100 consists of lignin or lignin derivatives, biodegradable polyester from natural sources, lignocellulose fibres, natural resins and waxes. The main parameters of both materials are given in Table 1.

### 2.2. Coating of the Commercial Flax Yarn and Parameters

**Figure 1:**
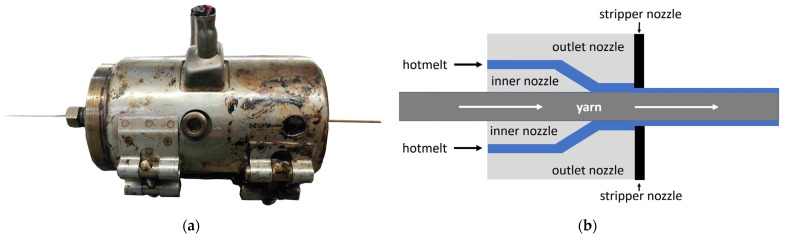
(**a**) Picture of the sheathing nozzle; (**b**) functional sketch of the sheathing nozzle.

Extrusion coating was carried out on a Thermo Scientific Eurolab 16 twin screw extruder (Figure 1a) with the software Prism Eurolab 16 from Thermo Fisher Scientific, Karlsruhe, Germany. In the twin-screw extruder, seven conveying zones can be heated independently of each other. In the case of temperature-sensitive thermoplastics, the melting process from the inlet to the nozzle can be precisely adjusted. The coating process was carried out horizontally at the head of the extruder through a modified sheathing nozzle. This sheathing nozzle consists of three individual nozzles (inner nozzle, outlet nozzle, stripper nozzle) with different tasks (Figure 1b). The yarn to be coated passes through an inner nozzle with an adjusted diameter and is coated with the molten matrix material by the outlet nozzle. The coated yarn is given its final diameter in a final stripping process with a stripper nozzle. 

The yarn material used was a commercially available flax yarn (FL commercial yarn) and were coated with thermoplastic lignin. Before being used the thermoplastic granules were dried in a drying oven at 50 °C for 4 h. The die diameters were adapted to the yarn in the preliminary tests. The following criteria were considered when choosing the nozzle diameter. The nozzle had to have just enough size that the yarn can pass through the inner nozzle without any resistance but not generate vibrations due to excess space. Initially, the inner nozzle diameter was reduced from 1.2 mm to 1.0 mm. The outlet nozzle applied the matrix coating material to the yarn. The final diameter of the coated yarn was brought down from 1.6 mm to 1.4 mm by a stripper nozzle at the end of the sheathing nozzle. A further reduction in the nozzle diameters was not possible because of increasing yarn breakages.

**Figure 2:**
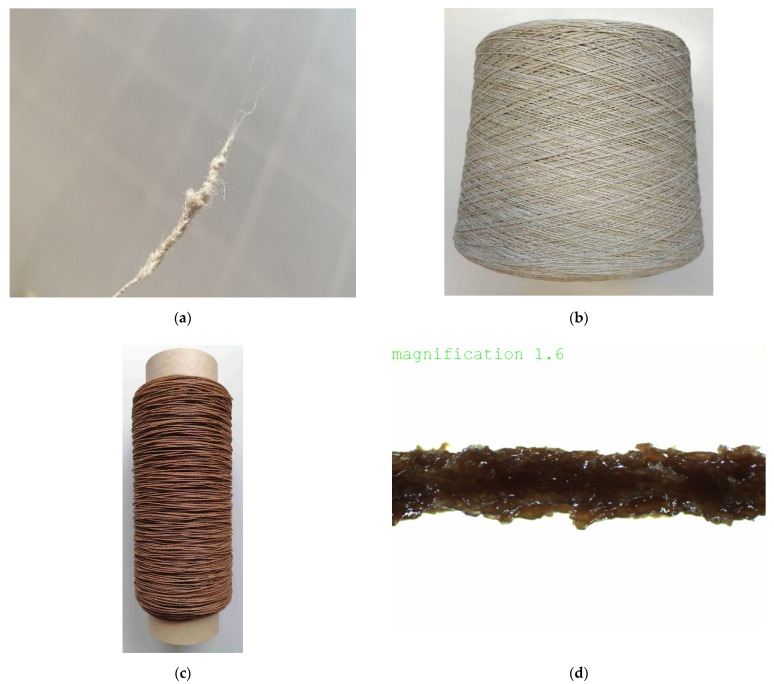
(**a**) Example of breakage in flax yarn; (**b**) bobbin of flax yarn (FL) without coating; (**c**) bobbin of flax yarn with lignin (FL-LI coated yarn); (**d**) close-up view of FL-LI coated yarn (the magnification is 1.6).

The selection of the die diameter in conjunction with the temperature and pressure setting in the extruder, as well as the throughput speed (production speed) proved to be very demanding. The compliance with the very narrow temperature processing range of the thermoplastic coating material was equally challenging. The following two factors made a continuous yarn coating process difficult: the flax yarn to be coated had (1) many splices with larger diameters. These caused blockages in the sheathing nozzle and ultimately yarn breaks and a production stop (Figure 2a). The other problematic factor was (2) the very narrow temperature range of the thermoplastic coating material. There are differences in the production pressure and in the viscosity behaviour in a range from 180 °C to 184 °C. Especially when the ideal processing temperature of 182 °C was briefly exceeded, deposits formed in the sheathing nozzle due to thermoplastic segregation.

Figure 2b show the uncoated yarn bobbin, while Figure 2c depicts the coated flax yarn (1600 m), which was produced with the settings given in Table 2 and Table 3. Figure 2d reveals in a close-up view the sawtooth-like structure of the lignin coated flax yarn (FL-LI coated yarn).

### 2.3. Weaving Process and Parameters

The warping process of the lignin coated flax yarn took place on the mini warping machine SW550, from the CCI Tech Inc., New Taipei City, Taiwan. The FL-LI coated yarn had to be used as the warp because of the stiffness and strength of the yarn. The yarn cannot be cut and is therefore not suitable as weft yarn. During the warping process the tension and the speed had to be adjusted to avoid yarn breakages. The speed during the warping process had to be chosen low, as the yarn is very stiff. The tension during the warping process too had to be chosen low. This eventually means that the warp tension of the individual threads is inhomogeneous, but it prevents the brittle FL-LI coated yarn from breaking. To avoid damaging during the warping process, the yarn was fed with a positive creel, CCI Tech Inc., New Taipei City, Taiwan. The warp length was 3.60 m because of the mechanical limitations of the mini warping machine SW550.

**Figure 3:**
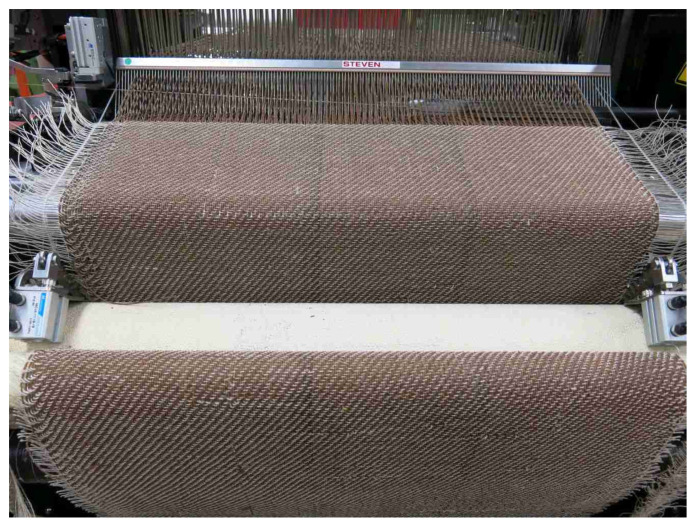
Weaving with FL-LI coated yarn (warp) and FL commercial yarn (weft).

The FL-LI coated yarn was woven on the sample weaving loom Evergreen II (Figure 3) form CCI Tech Inc., New Taipei City, Taiwan. The weaving parameters are given in Table 4. 

**Figure 4:**
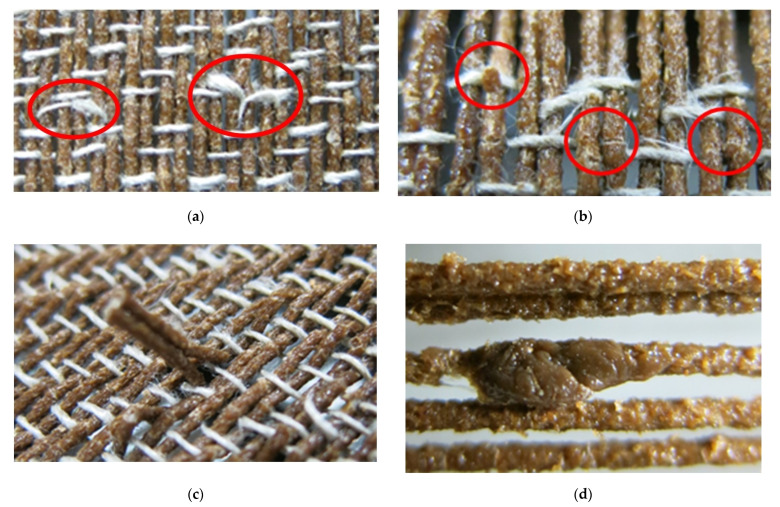
(**a**) Broken weft yarn; (**b**) broken lignin coating of the warp yarns; (**c**) broken Warp yarn; (**d**) in-homogeneous lignin coating of the warp yarn.

The weft insertion speed, and the raising and lowering speed of the shafts had to be set to as slow as possible due to the roughness and sharpness of the FL-LI coated yarn. The faster the weft insertion speed, the more likely the weft yarn breaks as shown in Figure 4a. The lignin coating breaks along the last inserted weft when the shafts move, as seen in Figure 4b. The warp tension has to be raised because of the inhomogeneous warp beam. The raising of the warp tension further led to warp breaks due to the brittleness of the FL-LI coated yarn (Figure 4c). The roughness of the yarn resulted in abrasion of the lignin coating, and high wear of the weaving reed and the weaving healds. Another problem was the partially inhomogeneous lignin coating on the yarn Figure 4). The partially inhomogeneous lignin coating on the yarn led to yarn breakages due to unsteady yarn tension and the tangling of the yarn in the healds. The length of the finished fabric was 2.70 m.

### 2.4. Composite Fabrication

Samples measuring 400 mm × 400 mm were cut out from the flax lignin fabric and pre-dried for 2 h at 80 °C in an oven. Afterwards the fabric pieces were stacked in three layers and consolidated in a VCP500 vacuum hot press from Maschinenfabrik Lauffer GmbH & Co. KG, Horb, Germany. In all layers, warp (FL-LI coated yarn) and weft (FL commercial yarn) yarns were aligned in the same direction. Two sheets were produced for the extraction of specimens according to the two directions warp and weft.

**Figure 5:**
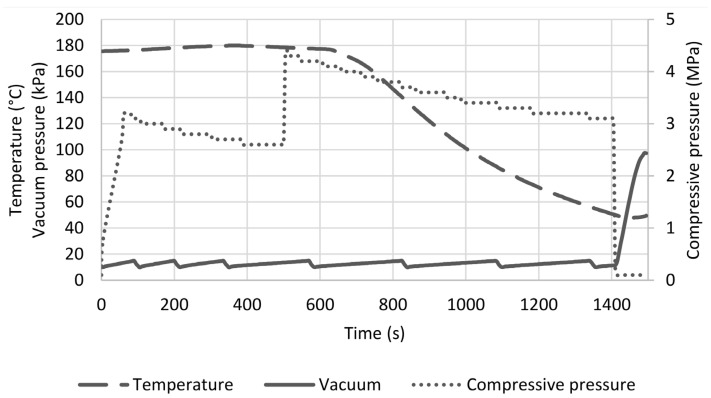
Hot pressing cycle of FL-LI composite plates.

**Figure 6:**
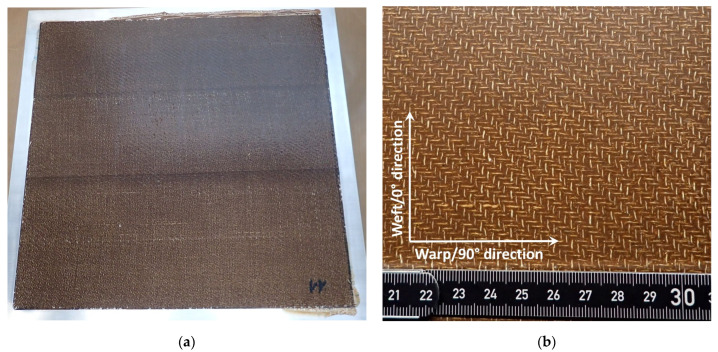
(**a**) Consolidated FL-LI composite plate with aluminium press frame; (**b**) warp (90°) and weft (0°) direction of composite plate.

The same pressing parameters, which were determined and applied in [33], were used. They are given in Table 5. Figure 5 shows the pressing cycle for both flax-lignin composite plates (FL-LI composite). Temperature and vacuum pressure are plotted on the left axis and compressive pressure on the right axis. One of the composite plates can be seen in Figure 6a. The weft (0°) and warp (90°) directions are given in Figure 6b.

### 2.5. Characterisation

#### 2.5.1. Determination of Yarn Coating

The determination of the yarn coating add-on is conducted gravimetrically based on DIN EN ISO 2060 textiles—yarn from packages—determination of linear density (mass per unit length), by the skein method. A reeling machine from Zweigle Textilprüfmaschinen GmbH & Co. KG, Reutlingen, Germany, was used to remove 2 times 50 m of yarn from a bobbin. Then, the obtained skeins of yarns were weighed with a scale from Mettler Toledo PM 200, Gießen, Germany.

To calculate the add-on, the mean value was determined from two skeins of 50 m yarns. From the difference value of the weight of the uncoated yarn and the coated yarn, the coating add-on was calculated in per cent. 

The tests were conducted at a temperature of 20 °C ± 2 °C and relative humidity of 65% ± 2% (according to DIN EN ISO 139) on samples conditioned for 24 h at standard climate.

#### 2.5.2. Tensile Properties of Yarn

The tensile tests of the FL commercial yarn and FL-LI coated yarn were carried out according to DIN ISO 2062 with the universal testing machine Zwick 1455 ZMART.PRO from ZwickRoell GmbH & Co. KG, Ulm, Germany. The length of the yarn samples was 500 mm. The pre-tension force was set to 0.5 cN/tex and the test speed to 500 mm/min. The tensile force was recorded as a function of deformation. The scope of measurement was 20 samples and the average value for each type of yarn variant was determined.

The tests were conducted on samples conditioned for 24 h at standard climate (temperature of 20 °C ± 2 °C and relative humidity of 65% ± 2% according to DIN EN ISO 139).

#### 2.5.3. Fibre Mass Fraction and Fibre Volume Fraction at Absolute Dry Weight

The standard DIN EN ISO 1833-1 describes the extraction of bast fibres from cellulose-free components from textiles and textile products using sodium hydroxide. To determine the mixing ratio at absolute dry weight, 1 g of the sample was dried with a weighed filter crucible for 16 h at 105 °C ± 3 °C in a drying oven and weighed after cooling. Sodium hydroxide solution (1.5 mol/l solution) was boiled in a flask equipped with a re-flux condenser for at least 15 min and after separating the air from the solution (due to boiling), the sample was placed in the flask and boiling was continued for 1 h. Then the sample was dried and weighed after cooling. Afterwards, the sample was rinsed with water by continuous suction for at least 5 min and immersed in an acetic acid solution of 0.1 mol/l for 10 min. The contents of the flask were then filtered, rinsed with water until neutralised and dried, after which they were weighed. Two samples (double determination) per process step were tested.

Based on the mass fractions and the known densities (flax fibre = 1.5 g/cm³), the volume fraction of the natural fibre was calculated for the lignin granule, coated yarns, woven fabrics and the bio-based composites.

#### 2.5.4. Analysis of Mechanical Properties of Bio-Based Composite

Using a Z100 universal testing machine from ZwickRoell GmbH & Co. KG, Ulm, Germany, with special wedge grips and an external optical strain gauge, tensile tests were performed according to DIN EN ISO 527-5. The dimensions of the specimens were 250 mm × 25 ± 0.2 mm × 2.3 ± 0.2 mm. The preload was set to 0.1 MPa and the test speed to 1 mm/min. Six measurements were made in which the tensile force was recorded as a function of deformation and the average value was considered. The stress-strain behaviour was evaluated with the software testXpert^®^II (version 3.71).

With a Zwick Z020 from ZwickRoell GmbH & Co. KG, Ulm, Germany, the bending properties of the manufactured composite were determined in a 4-point bending test (method B) once in warp and once in weft direction according to the standard DIN EN ISO 14125. The specimen measurement and the automatic calculation of the cross-sectional area-specific bending modulus were carried out using testXpert^®^II software (version 3.71). The radius of the loading and support member was 2 ± 0.2 mm. The preload was set to 0.2 MPa and the test speed to 2 mm/min. For each direction, 6 specimens with the dimensions 60 ± 0.2 mm × 15 ± 0.2 mm × 2.3 ± 0.2 mm were tested.

All tests were carried out in accordance with the standard DIN EN ISO 139 at standard climate (temperature of 20 ± 2 °C and relative humidity of 65 ± 2%) on specimens conditioned for 24 h.

## 3. Results and Discussion

### 3.1. Yarn Properties

The add-on of the lignin coated yarn was 336%, and therefore 111% over the desired add-on of 225%. The desired add-on was calculated to achieve a fibre mass fraction of 50%. Due to the processing properties of the lignin granulate, the short processing time, the temperature sensitivity during the coating process and therefore low possibility to vary the viscosity, a higher add-on was received. Additionally, the diameter of the stripper nozzle was responsible for the outer diameter of the coated yarn. After testing several nozzle diameter variants, it was found that an even coating could only be achieved with a stripper nozzle of 1.4 mm diameter.

**Figure 7:**
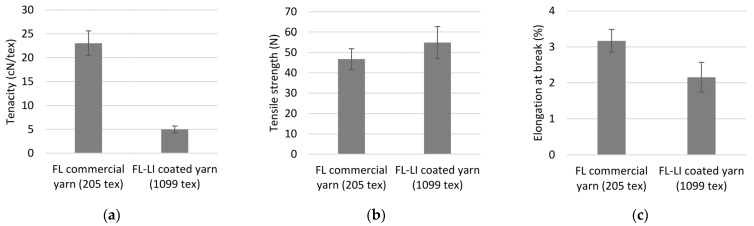
(**a**) Fineness-related tensile strength of yarns; (**b**) tensile strength of yarns (**c**) elongation at break of yarns.

The mechanical properties of the FL-LI coated yarn and the FL commercial yarn are given in Table 6 and in Figure 7. The yarn count increases approximately fivefold from 205 tex for FL commercial yarn to 1099 tex for the coated yarn due to the high proportion of lignin in the process. The reinforcing fibre content of the FL-LI coated yarn is thus 24.88 wt.%. This difference can also be seen in the fineness-related maximum tensile strength between coated flax lignin and FL commercial yarn. Through the lignin coating and the thus reduced proportion of reinforcing fibres, the strength of the entire yarn is reduced by about 78% from 23.06 cN/tex to 5.00 cN/tex. A comparison of the absolute values of the maximum tensile strength shows an increase from 46.72 N for the uncoated yarn to 54.92 N for the coated yarn. The influence of the rather brittle lignin coating can also be seen in the values of elongation. The elongation is reduced from 3.17% for the FL commercial yarn to 2.16% for the FL-LI coated yarn.

### 3.2. Fabric Properties

The warp beam production faced difficulties due to the stiffness and brittleness of the yarn, and resulted in an inhomogeneous warp beam. In Table 7, the properties of the woven fabric are given. The grammage values are calculated from 400 mm × 400 mm pieces. In the experimental study, the weft yarn density with five threads per cm was found to be the best choice. A low weft insertion speed at three picks per minute was chosen, because of the sharpness of the warp yarn as the sawtooth-like structure can damage the weft yarn.

### 3.3. Fibre Volume Fraction

The fibre volume fractions (FVF) of the semi-finished products for the process steps from the initial material (lignin granulates; 14.32 vol.%) to yarn (23.38 vol.%), fabric (34.47 vol.%) and finally to the composite material (30.81 vol.%). Due to the very high proportion of matrix material in the yarn due to the process, instead of a unidirectional (UD) fabric, a bidirectional one was produced. The coated yarn was used as warp yarn and the FL commercial yarn as weft yarn, thus increasing the FVF by about 10 vol.%. The drop in FVF of 3.66 vol.% between the fabric and the composite is particularly noticeable. Since no loss of fibre material could be detected during the consolidation process, the cause could possibly lie in the test method used according to DIN EN ISO 1833-1. Even though the standard is specifically for the extraction of bast fibres from cellulose-free components, the result suggests that in addition to the lignin, components of the flax fibres are also decomposed. It is therefore conceivable that due to the time required to dissolve the consolidated composite compared to the non-consolidated coated yarn or woven fabric, additional components such as hemicelluloses, lignin, pectin and/or waxes are dissolved out of the bast fibres, thereby reducing the FVF of the composite.

### 3.4. Composites

The bidirectional fabric was cut to size, stacked into three layers each and consolidated into a total of two composite sheets. One was examined in the 0° direction (weft yarn; FL-LI 0°) and one was examined in the 90° direction (warp yarn; FL-LI 90°) with regard to its mechanical properties. In addition, the characteristic values of a unidirectional flax-polylactide composite (FL-PLA UD composites) were compared for classification, as no composites made from coated natural fibre yarns could be found in the literature [33]. With the exception of the tensile and flexural elongation, the difference in mechanical properties between bidirectional and quasi-unidirectional fabric is clearly evident.

#### 3.4.1. Tensile Properties

**Figure 8:**
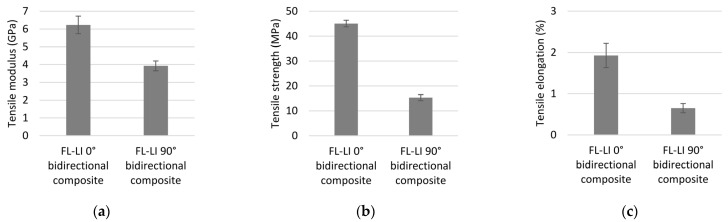
(**a**) Tensile modulus of FL-LI composites; (**b**) tensile strength of FL-LI composites; (**c**) elongation of FL-LI composites.

**Figure 9:**
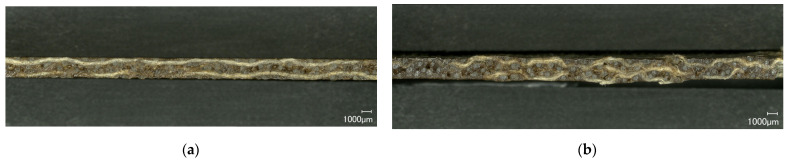
(**a**) Cross section showing the course of warp yarn of the FL-LI composite; (**b**) cross section showing the course of weft yarn of the FL-LI composite.

**Figure 10:**
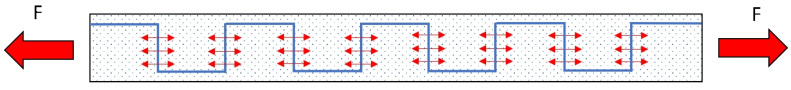
Load transfer directions of the weft yarn during the tensile test with idealised yarn course. The red arrows indicate the force (F). The blue line shows the weft yarn in the matrix background.

**Figure 11:**
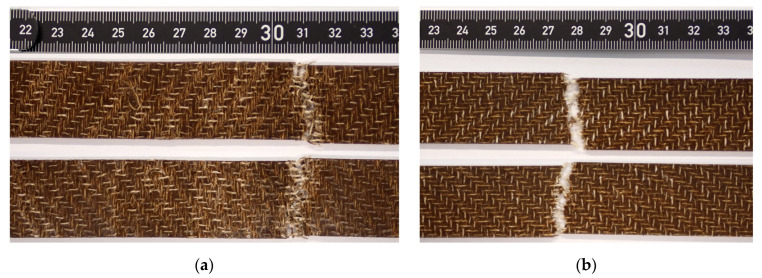
Fracture zones of selected tensile test specimens (**a**) in 0° (weft direction, above front view and below back view); (**b**) in 90° (warp direction, above front view and below back view).

**Figure 12:**
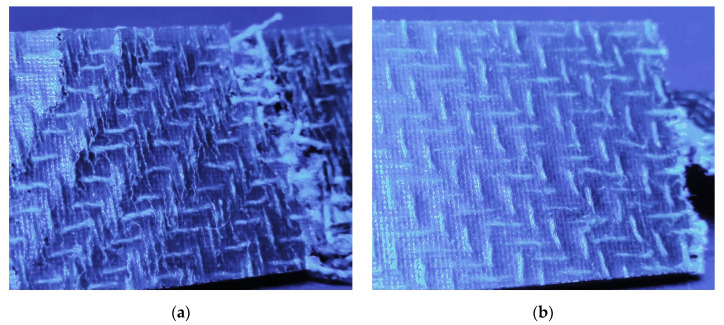
Blue coloured detailed view on the structure of the fracture zones of selected tensile test specimens:(**a**) tensile test in weft direction with plastic deformation along the diagonal twill weave lines; (**b**) tensile test in warp direction without plastic deformation.

The results of the tensile test according to DIN EN ISO 527-5 are shown in Figure 8a–c. The values for FL-LI 0° (weft yarn) for tensile modulus (6.23 GPa), tensile strength (45.06 MPa) and elongation (1.93%) are higher than those of FL-LI 90° (warp yarn; 3.92 GPa, 15.31 MPa, 0.65%). Due to 6 and 5 yarns per cm in in warp and weft directions, respectively, approximately equal, if not opposite characteristic values were expected. The reason for the higher tensile modulus, the higher tensile strength values and the higher tensile elongation were found to be the yarn course of warp-weft system in the fabric. The FL-LI yarns of the warp system laid stretched in the fabric, because they are very stiff due to their coating and have therefore, hardly any undulation after the weaving process (Figure 9a). In the weft direction, the yarn undulates over and under the warp threads and has a meandering yarn course in the fabric (Figure 9b). Due to the undulations, the weft yarn in the composite is longer and has thereby a larger contact area with the matrix system as well as a larger clamping length than the warp yarns. When the consolidated sample is subjected to tension during the tensile test, the weft yarns can therefore absorb more load (Figure 10). The meandering yarn course in the weft direction is also helpful against the weft yarns being pulled out of the matrix. The higher elongation can also be explained by the increased load transfer from the weft yarns to the matrix system. The undulation of the weft yarns is reduced during the tensile test in 0° direction, which results in a plastic deformation of the sample along the diagonal twill weave lines (Figure 11a and Figure 12a), compared to the tensile test in 90° with straight warp yarns where no plastic deformation takes place (Figure 11b and Figure 12b).

#### 3.4.2. Flexural Properties

**Figure 13:**
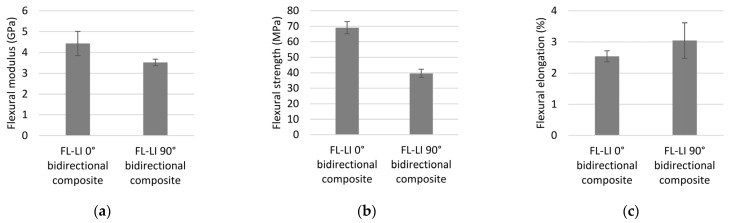
(**a**) Flexural modulus of FL-LI composites; (**b**) flexural strength of FL-LI composites; (**c**) elongation of FL-LI composites.

The bending properties of the FL-LI composites were determined in a 4-point bending test according to DIN EN ISO 14125 and are shown in Figure 13a–c. The flexural modulus of elasticity of FL-LI 90° is only slightly lower at 3.52 GPa compared to that of FL-LI 0° at 4.43 GPa. Due to higher yarn density in warp direction (6 yarns per cm) compared to that in the weft direction (5 yarns per cm) of the FL-LI fabric, a higher flexural modulus in the warp direction (90°) was expected than in the weft direction (0°). However, the 4-point bending tests proved a higher flexural modulus in the weft direction. The higher flexural modulus in the weft direction (0°) is due to the fact that the weft yarns are located further outside the central neutral bending axis, in the FL-LI composite, than the warp yarns (90°). Below the neutral bending line, the yarns are subjected to tensile stress, and above it, to compressive stress. Thus, the weft yarns, which are further away from the neutral bending line are significantly more stressed during the 4-point bending test. The higher flexural modulus in the weft direction is therefore mainly generated by the weft yarns below the neutral bending line.

The same effects as for the flexural modulus are evident in the flexural strength tests. The FL-LI composite has a higher flexural strength in the weft direction (69.05 MPa) than that in the warp direction (39.63 MPa). The weft yarns, which are located more off-centre than the warp yarns in the composite, generate a higher section modulus than the warp yarns. The further the weft yarns are displaced under the neutral bending line of the FL-LI composite, the greater their influence on a higher flexural strength.

Elongations of 2.54% and 3.05% are achieved in the weft and warp directions, respectively. The more even distribution of the warp yarns in the FL-LI composite increases the elongation in the warp direction, because the warp yarns are closer to the neutral bending line than the weft yarns. In contrast to the weft yarns, the yarns in the warp direction are not stressed as much in the 4-point bending test. Therefore, the warp yarns only break at a larger deflection.

## 4. Conclusions

This paper presents an experimental study on the use of lignin as a matrix material and as a coating of yarns as an alternative for the semi-finished production of composite components. A commercial flax yarn is coated with a lignin matrix using an extrusion process. This coated yarn was processed into a woven fabric and consolidated into composite panels. The semi-finished products thus produced, yarn and composite material, were examined and analysed with regard to their characteristic properties.

The coating by thermal extrusion of flax yarn with lignin was successful. The lignin product passes quickly from the liquid to the solid aggregate state and make the yarn very stiff. Temperature adjustments in the individual extruder zones, as well as varying the production speed result in an acceptable but not satisfactory coating process. To increase uniformity of the coated yarn in future work, a yarn without splices will be used to prevent yarn breakage during the coating process. Furthermore, the lignin formulation has to be improved regarding a longer processing time, a wider temperature processing range and for a smooth surface of coated yarn in addition with reduced add-on. A coating degree of 336% was attained. However, this could not be brought down to the desired 225%.

The coated flax yarns exhibited moderate elongation behaviour and good strength with a fibre volume content of nearly 35 vol.%. These properties allow for further processing by weaving, but the low flexibility transverse to the yarn axis and the saw wire-like surface structure posed a considerable challenge. 

The fabrics were produced with the lignin coated flax yarn (1099 ± 15 tex) in the warp direction and the commercially flax yarn (205 ± 5 tex) in the weft direction. The warp and weft densities are 6 and 5 yarns per cm, respectively. The lignin coated yarn is very brittle and therefore, gentle and slow processing are required. Nevertheless, the processing of the coated yarn remains intricate. To minimise damage to the yarn, the weaving shed should be reduced in size and the warp tension should be as low as possible. The width of the woven fabric is 500 mm. Another problem is the sharp-edged nature of the lignin coated flax yarn. This causes increased wear on the healds and the weaving reed. Large healds and a coarser weaving reed could bring improvement.

The analysis of the composite material with regard to the tensile and bending proper-ties showed clear differences between the weft (FL-LI 0°) and the warp (FL-LI 90°) direction, which can be attributed to the position and orientation of the flax yarns in the warp and weft directions in the composite. In addition, investigations are needed on the general fibre-matrix adhesion between flax yarn and lignin as well as on the influence of the extrusion process on the fibre-matrix adhesion. Furthermore, the penetration depth of the lignin matrix into a compact structure, such as the flax yarn, has to be analysed. 

To summarise, the use of lignin as a matrix offers a possibility for the cascading use of biomass, in which the use of by-products or waste products contributes to closing material cycles instead of their purely thermal utilisation, and leads to new advanced bio-based materials. Coating of (natural fibre) yarns with lignin presents an alternative way to manufacture hybrid yarns, which can be used to produce semi-finished products for composite components.

## Figures and Tables

**Table 1:** Materials used.

Parameter	Commercial Flax Yarn	Lignin Granule
Manufacturer	N.V. JOS VANNESTE S.A.	TECNARO GmbH
Product name	Flax yarn	ARBOFORM^®^ L V100
Fineness	205 tex ± 5 tex	
Density		1.29 g/cm³
Melt volume rate (190 °C/2.16 kg)		30 cm³/10 min

**Table 2:** Parameters of extruder for production of FL-LI coated yarn.

Production Parameters	Value
Nozzle diameter inside	1.0 mm
Nozzle diameter outside	1.4 mm
Winding speed	10 m/min
Delivery volume	6.98 g/min

**Table 3:** Temperature zones of the extruder.

Temperature Zone	Temperature
Inlet (funnel)	178 °C
Zone 1	182 °C
Zone 2	182 °C
Zone 3	182 °C
Zone 4	180 °C
Zone 5	180 °C
Head (nozzle)	180 °C

**Table 4:** Weaving parameters.

Weaving Parameters	Value
Warp material	Lignin coated flax yarn (FL-LI coated yarn) 1099 ± 15 tex
Warp density	6 yarns/cm
Warp tension	25 cN
Weft material	Flax yarn (FL commercial yarn) 205 ± 5 tex
Weft density	5 yarns/cm
Weft insertion speed	3 picks/min
Width	0.50 m
Type of fabric	Twill 2/2

**Table 5:** Pressing parameters.

Pressing Parameter	Value
Temperature	180 °C
Holding time	10 min
Vacuum	10 kPa
Compression pressure	3.1 MPa
Pre-drying temperature	80 °C
Pre-drying time	120 min
Venting phase	None

**Table 6:** Linear density and fibre mass fraction of yarns.

Yarn	Linear Density	Natural Fibre Mass Fraction
FL commercial yarn	205 ± 5 tex	100%
FL-LI coated yarn	1099 ± 15 tex	24.88%

**Table 7:** Textile properties of the woven fabric.

Woven Fabric	Grammage	Fibre Mass Fraction	Fibre Volume Fraction
FL-LI twill 2/2	894.31 g/m²	34.47%	30.81%

## Data Availability

Data are contained within this article.
